# Claudin-1 Is a Valuable Prognostic Biomarker in Colorectal Cancer: A Meta-Analysis

**DOI:** 10.1155/2020/4258035

**Published:** 2020-08-14

**Authors:** Didi Zuo, Jiantao Zhang, Tao Liu, Chao Li, Guang Ning

**Affiliations:** ^1^Department of Endocrinology and Metabolism, The First Hospital of Jilin University, Changchun, China; ^2^Department of Colorectal and Anal Surgery, The First Hospital of Jilin University, Changchun, China; ^3^Department of Endocrine and Metabolic Diseases, Rui-Jin Hospital, Shanghai Jiao Tong University School of Medicine, Shanghai, China

## Abstract

**Background:**

Claudin-1 plays an important part in maintaining the mucosal structures and physiological functions. Several studies showed a relationship between claudin-1 and colorectal cancer (CRC), but its prognostic significance is inconsistent. This meta-analysis assessed the prognostic value and clinical significance of claudin-1 in CRC.

**Materials and Methods:**

We retrieved eligible studies from PubMed, Cochrane Library, Embase, and Web of Science databases before February 10, 2020. The hazard ratio (HR) with 95% confidence interval (CI) was applied to assess the correlation between claudin-1 and prognosis and clinical features. Heterogeneity was assessed by the Cochran *Q* test and *I*-square (*I*^2^), while publication bias was evaluated by the Begg test and Egger test. Test sequence analysis (TSA) was used to estimate whether the included studies' number is sufficient. The stability of the results was judged by sensitivity analysis. Metaregression was utilized to explore the possible covariance which may impact on heterogeneity among studies.

**Results:**

Eight studies incorporating 1704 patients met the inclusion criteria. Meta-analysis showed that the high expression of claudin-1 was associated with better overall survival (HR, 0.46; 95% CI, 0.28–0.76; *P* = 0.002) and disease-free survival (HR, 0.44; 95% CI, 0.29–0.65; *P* = 0.003) in CRC. In addition, we found that claudin-1 was related to the better tumor type (*n* = 6; RR, 0.60; 95% CI, 0.49–0.73; *P* < 0.00001), negative venous invasion (*n* = 4; RR, 0.81; 95% CI, 0.70–0.95; *P* = 0.001), and negative lymphatic invasion (*n* = 4; RR, 0.83; 95% CI, 0.74–0.92; *P* = 0.0009).

**Conclusion:**

The increased claudin-1 expression in CRC is associated with better prognosis. In addition, claudin-1 was related to the better tumor type and the less venous invasion and lymphatic invasion.

## 1. Introduction

Colorectal cancer (CRC) is the third most common malignant tumor all of the world. There were 1.4 million new CRC cases every year [1]. It is expected to increase by 60% to 2.2 million new CRC cases and 1.1 million deaths in ten years [2]. The treatments include surgery, chemotherapy, and radiotherapy. Over the past 30 years, effective screening measures and multimodal therapies had depressed the incidence and the mortality rate and improved long-term survival rate. The incidence of CRC had decreased approximately 3% per year between 2003 and 2012 [3, 4]. In high-income countries, 5-year relative survival has reached almost 65%, but in low-income countries, it remained less than 50% [5–7]. Tumor stage is the most important criterion for judging prognosis and guiding treatment. At present, the domestic and internationally recognized standards for CRC staging are the TNM and the improved Dukes staging method developed by the International Union Against Cancer (UICC) and the American Cancer Society (AJCC). However, the current Dukes or TNM staging cannot monitor tumor progression dynamically and reflect the metastasis accurately. Recently, new prognostications were identified and played an important role in CRC, like biologic, genetic, and other molecular information [8–11].

Claudins are the major components of tight junctions (TJs), a kind of transmembrane proteins, and localize at the apex of epithelial cells in the colon [12, 13, 14]. In normal colon tissue, claudin-1 participates in maintaining the mucosal barrier structure and normal physiological functions [15, 16], regulating the permeability of the intestinal mucosal barrier, and preventing harmful macromolecular substances from entering the intestine. In recent years, the complex function of claudin-1 in tumors was unraveled by analyzing the expression of claudin-1 in colorectal adenocarcinoma and normal mucosa. Many studies have shown that the abnormal expression of claudins is related to the tumor development and prognosis, such as prostate cancer [17], gallbladder cancer [18], breast cancer [19, 20], esophageal adenocarcinomas [21], gastric adenocarcinoma [22], laryngeal carcinoma [23], lung cancer [24–26], and glioblastoma [27].

However, the prognostic value and clinical significance of claudin-1 are controversial [28]. Some studies have shown that the decreased expression of claudin-1 indicates worse prognostic and aggressive tumor behaviors [29] and linked with higher histological grade, invasion depth, and lymph invasion in CRC [30, 31]. However, other studies showed that there is no relation between them [32]. Therefore, we performed this meta-analysis to investigate the prognostic and clinical significance of claudin-1 expression in CRC.

## 2. Materials and Methods

### 2.1. Data Sources and Search Strategy

This study was based on Preferred Reporting Items for Systematic Reviews and Meta-analyses (PRISMA) guideline (File S1). We retrieved articles published before February 18, 2020, in PubMed, Embase, the Cochrane Library, and Web of Science databases using medical subject headings (MeSH) and their free-text words. Search terms include “Colorectal Neoplasm”/“Colorectal Tumor”/“Colorectal Carcinoma”/“Colorectal Cancer”/“colonic cancer”/“rectal cancer”/“crc”/“colon cancer”/“rectum cancer” and “claudin-1”/“claudin 1”/“CLDN-1”/“CLDN 1”(File S2). No ethical approval or patient consent was required in this study because this meta-analysis was based on previous studies and does not contain any studies with human or animal subjects.

### 2.2. Inclusion and Exclusion Criteria

Inclusion criteria were as follows: (1) the study belonged to a cohort study; (2) the study object were patients with colorectal cancer; (3) the study content was the relationship between claudin-1 expression and CRC survival rate; and (4) the outcome of the study is the survival rate of colorectal cancer. These studies were excluded if they (1) were duplicate publications or overlapping studies; (2) exclusively used animals or cell lines; (3) were case reports, reviews, conference reports, abstracts, books, or letters; or (4) do not have enough data to assess the correlation between claudin-1 and survival outcome.

### 2.3. Data Extraction and Quality Assessment

There were two independent authors who extracted and summarized data. Any disagreement was settled by the adjudicating senior authors until consensus was reached. We extracted the following information: first author, publication year, country, number of patients, tumor site, mean age, TNM stage, follow-up time, claudin-1 expression, detection method, antibody, cutoff value for claudin-1, claudin-1 expression rate, survival outcome, Newcastle Ottawa Scale (NOS), and method for extracting survival data. If there were hazard ratios (HRs) in the article, we extract it directly. If HRs were not provided directly, we used the software Engauge Digitizer Version 4.1 to extract Kaplan-Meier curve data and calculated HRs. We assessed the quality of eligible studies by NOS [33].

### 2.4. Statistical Analysis

We used Preferred Reporting Items for Systematic Reviews and Meta-analyses (PRISMA) guidelines in this meta-analysis [34]. Hazard ratio (HR) and relative risk (RR) with corresponding 95% confidence interval (CI) were applied to evaluate the correlation between claudin-1 expression levels and prognosis (OS/DFS) and clinical characteristics of CRC [35]. The Cochran *Q* test and *I*^2^ test were used to evaluate the impact of study heterogeneity on the results of the meta-analysis [36]. Based on the Cochrane review guidelines, *I*^2^ > 50% indicates severe heterogeneity and the analysis should use a random effects model. Otherwise, the fixed effect models were utilized [37]. In addition, we performed metaregression analysis to explore the source of heterogeneity, Begg's and Egger's tests to detect publication bias [38, 39], trial sequential analysis (TSA) to estimate whether the sample sizes required for the meta-analysis were sufficient [40, 41], and sensitivity analysis by excluding one study at a time to confirm the robustness of the results. All statistical analyses were carried out by STATA (version 14.0, Stata Corporation, College Station, TX, USA) and Review Manager (version 5.3, Cochrane Collaboration, Copenhagen, Denmark).

## 3. Results

### 3.1. Characteristics of Enrolled Studies

**Figure 1:**
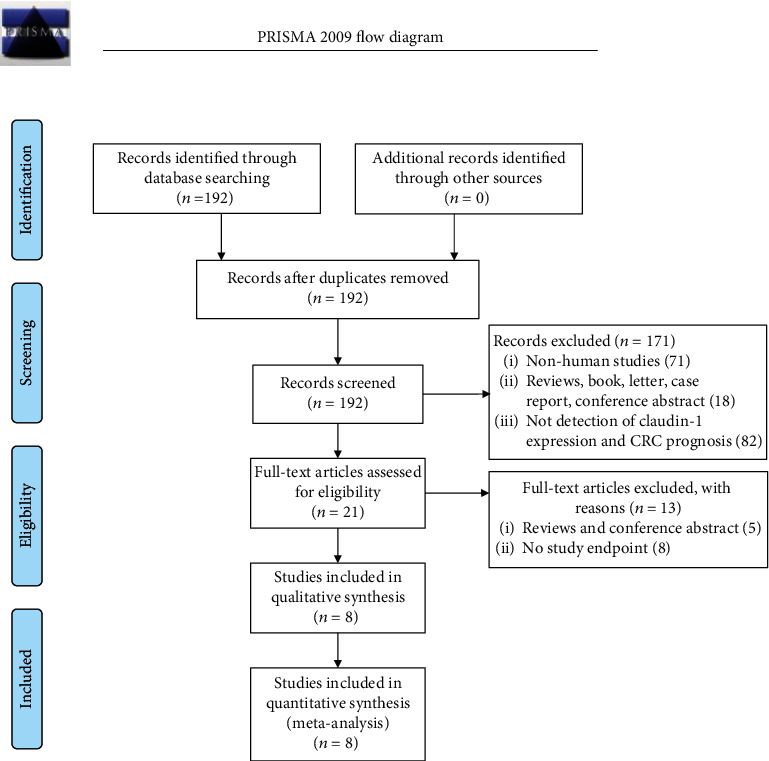


Figure 1 summarizes the flow diagram of the literature searching, and Table 1 shows the detailed characteristics of the eligible studies. Among the 192 articles that were retrieved, 171 records were excluded after screening the titles and abstracts. Among the other 21 articles, 13 articles were excluded, including reviews and conference (5) and lack of study endpoint (8). Thus, a total of eight studies were eventually included in this study [42–49]. The sample size was 1704 totally and ranged from 119 to 344 patients. The included studies were conducted in the USA, Japan, Korea, Turkey, and Canada and were published between 2005 and 2019. The NOS assessment for all studies is shown in Table S1, indicating the studies were of high quality.

### 3.2. Claudin-1 Expression and Survival Rate

**Figure 2:**
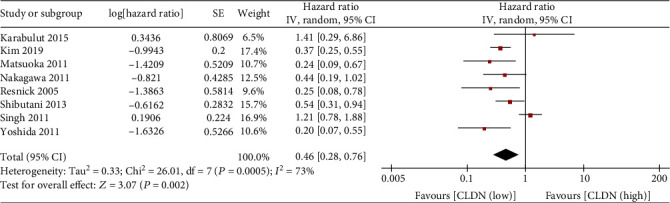


A total of eight studies explored the relationship between claudin-1 expression and OS. Because of the significant heterogeneity (*I*^2^ = 73%, *P* = 0.0005), random effects model was employed for evaluation. Our data indicated that the high expression of claudin-1 was associated with better OS (HR, 0.46; 95% CI, 0.28–0.76; *P* = 0.002; Figure 2).

**Figure 3:**
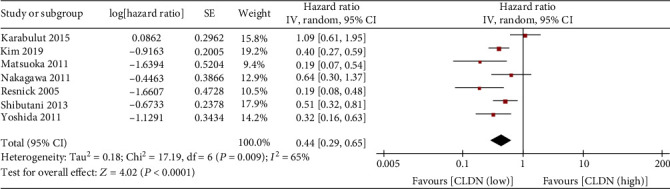


There were seven studies that reported the relationship between claudin-1 expression and DFS. Due to the significant heterogeneity (*I*^2^ = 65%, *P* = 0.009) between these studies, a random effects model was applied for meta-analysis. Our data indicated that the high expression of claudin-1 was associated with better DFS (HR, 0.44; 95% CI, 0.29–0.65; *P* < 0.0001; Figure 3).

### 3.3. Claudin-1 Expression and Clinical Features

Table 2 summarizes the relationship between claudin-1 expression and clinicopathological characteristics. The high expression of claudin-1 was significantly related to the better tumor type (*n* = 6; RR, 0.60; 95% CI, 0.49–0.73; *P* < 0.00001), negative venous invasion (*n* = 4; RR, 0.81; 95% CI, 0.70–0.95; *P* = 0.001), and negative lymphatic invasion (*n* = 4; RR, 0.83; 95% CI, 0.74–0.92; *P* = 0.0009). In addition, meta-analysis showed that claudin-1 was associated with early tumor stage (*n* = 3; RR, 0.75; 95% CI, 0.54–1.04; *P* = 0.09) and negative lymph node metastasis(*n* = 4; RR, 0.91; 95% CI, 0.82–1.02; *P* = 0.10), although it was not statistically significant.

Besides, we did not observe correlations between claudin-1 expression and other clinicopathological features, including perineural invasion (RR, 0.57; 95% CI, 0.25–1.31; *P* = 0.18; *n* = 2), depth of invasion (RR, 0.94; 95% CI, 0.72–1.22; *P* = 0.64; *n* = 4), lymph node metastasis (RR, 0.91; 95% CI, 0.82–1.02; *P* = 0.1; *n* = 4), distant metastasis (RR, 0.88; 95% CI, 0.67–1.15; *P* = 0.35; *n* = 2), size (RR, 0.88; 95% CI, 0.60–1.29; *P* = 0.52; *n* = 2), tumor site (RR, 1.01; 95% CI, 0.87–1.17; *P* = 0.92; *n* = 2), gender (RR, 1.02; 95% CI, 0.92–1.14; *P* = 0.66; *n* = 5), or age (RR, 1.15; 95% CI, 0.83–1.60; *P* = 0.33; *n* = 2).

### 3.4. Sensitivity Analysis and Publication Bias

**Figure 4:**
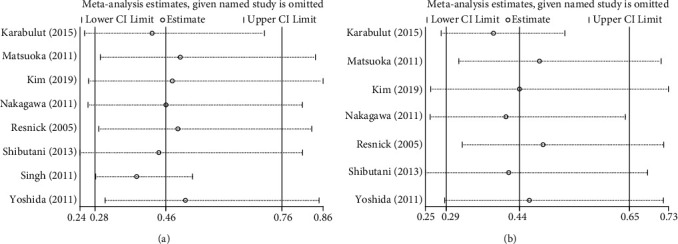


**Figure 5:**
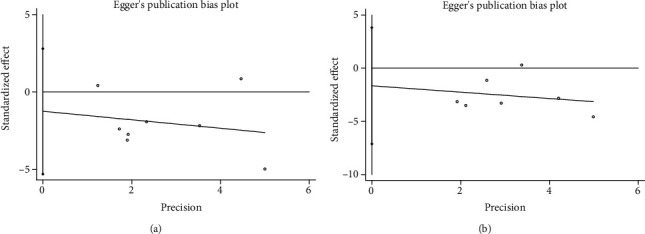


**Figure 6:**
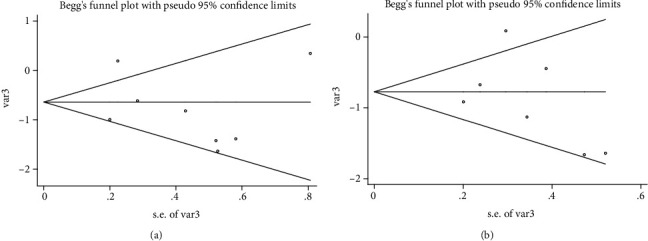


We performed sensitivity analysis by excluding one study at a time to confirm the robustness of the results for OS (Figure 4(a)) and DFS (Figure 4(b)). In addition, Egger's linear regression (OS, *P* = 0.480, Figure 5(a); DFS, *P* = 0.470, Figure 5(b)) and Begg's rank correlation test (OS, *P* = 1.000; Figure 6(a); DFS, *P* = 0.368, Figure 6(b)) showed that there was no publication bias in this study.

### 3.5. Trial Sequential Analysis and Metaregression Analysis

**Figure 7:**
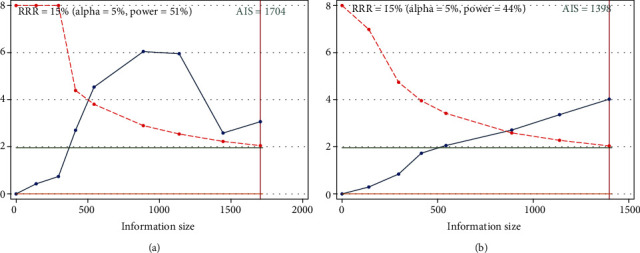


The cumulative *Z*-curve (blue line) reached the required information size (RIS) indicating that the number of cases included in this meta-analysis is sufficient. The blue line crosses the traditional threshold (horizontal line) and the TSA threshold (red line) indicating that the high expression level of claudin-1 was statistically significant with better OS (Figure 7(a)) and DFS (Figure 7(b)) of colorectal cancer.

We performed a metaregression analysis to explore possible sources of heterogeneity. The results showed that covariates (year, country, site, TNM stage, detection method, NOS score, and survival analysis) did not significantly affect OS and DFS of colorectal cancer (Table 3).

## 4. Discussion

CRC is one of the most common gastrointestinal tumors. There are approximately 1.2 million new CRC cases, and 600 000 die from the disease every year [50]. The prognosis of CRC has improved slowly but steadily during the past decades in the world. Early diagnosis and prognostic prediction are becoming more and more important for patients. This meta-analysis assessed the association between claudin-1 and prognosis, and the results showed that the high-expressed claudin-1 is correlated with lower aggressive tumor behavior and better prognosis (OS: HR, 0.46; 95% CI, 0.28–0.76; *P* = 0.002; DFS: HR, 0.44; 95% CI, 0.29–0.65; *P* < 0.0001).

This is an updated meta-analysis to clarify claudin-1 expression and prognosis in CRC. Previous meta-analysis by Jiang et al. showed that the claudin-1expression is associated with one, three, and five years of OS [51]. In comparison, the present analysis not only added an additional three studies [43, 44, 48] but also examined the HR for OS and DFS of CRC. We also included all claudin-1 detection methods, including ELISA and RT-PCR, which were excluded in the analysis by Jiang et al. In addition, new studies have emerged reporting on claudin-1 and CRC since the previous similar meta-analysis was published in 2017 [48, 52].

Since claudins were discovered, literature about the claudins' status and mechanisms during tumorigenesis is constantly expanding. Claudins in intestinal cytomembrane maintain the intestine's homeostasis; thus, abnormal expression of claudins may result in various pathophysiological conditions, and the loss of claudin-1 expression leads to tumor invasion and metastasis. In addition, NF-*κ*B was frequently activated in CRC tissue with low-expressed claudin-1.Some studies have shown that claudin-1 is upregulated in CRC, but this overexpression has a good prognostic value, overall survival, disease-free survival, less metastases, and less aggressive disease [45, 47]. Matsuoka et al. suggested that claudin-1 plays a part in tumorigenesis as a promoter, and the expression of this protein will be lost in tumor cells once the tumor is established and started the invasion process [45].

Since the clinical and pathological characteristics were often used to predict prognosis of CRC, the relationship between claudin-1 and the clinical and pathological characteristics of CRC was also discussed. The results indicate that claudin-1 expression is related to better tumor type, negative venous invasion, and negative lymphatic invasion. In addition, claudin-1 was associated with early tumor stage and negative lymph node metastasis, although not statistically significant (*P* = 0.09 and *P* = 0.10). It is well known that better tumor type, negative venous invasion, negative lymphatic invasion, early tumor stage, and negative lymph node metastasis were good indicators for predicting the prognosis of colorectal cancer. These data also confirm that high expression of claudin-1 can be used as a good prognostic biomarker for colorectal cancer. However, whether the relationship of high expression of claudin-1 with better prognosis is due to its relationship with better tumor type, negative venous invasion, and negative lymphatic invasion is not clear, which needs more studies to confirm it. In addition, we did not find any correlation between the expression of claudin-1 and nerve infiltration, depth of infiltration, lymph node metastasis, distant metastasis, tumor size, location, gender, and age due to the insufficient number of included studies.

Claudin-1 can be detected by immunohistochemistry in blood samples, which is a simple, easy, and feasible method. We can know well the progress, prognosis, and treatment effect of CRC by continuously monitoring the claudin-1 level. In addition, claudin-1 could guide us to formulate a reasonable treatment strategy. For example, we could choose different treatment strategies for resectable, unresectable, and potentially resectable tumors in advanced colorectal cancer patients. So, claudin-1 is of great significance in guiding the clinical treatment decisions and predicting prognosis.

There are some limitations in this study. Firstly, significant heterogeneity could be induced by the different detection methods and cutoff level. Secondly, all of the included studies were in English and observational studies, which contributed to selection bias and recall bias. Third, there may be some errors in the extraction of survival data from the Kaplan-Meier curve, which may affect the accuracy of the results. Finally, the robustness of the statistical results could be impacted by the sufficient number of eligible articles and patients.

## 5. Conclusion

This meta-analysis identified eight studies assessing the association between claudin-1 and prognosis and clinical characteristics of CRC. These studies suggest that the high expression of claudin-1 was associated with better overall survival and disease-free survival in CRC. Moreover, claudin-1 was related to the better tumor type, negative venous invasion, and negative lymphatic invasion. Overall, this meta-analysis showed that claudin-1 may be a valuable indicator for predicting prognosis and helping us accurately intervene in the progress of CRC. However, whether the relationship of high expression of claudin-1 with better prognosis is due to its relationship with better tumor type, negative venous invasion, and negative lymphatic invasion is not clear, which needs more randomized controlled trials (RCTs) to confirm the conclusion.

## Figures and Tables

**Table 1:** Main characteristics of the included publications.

First author	Year	Country	No. of patients	Site	Mean age (years)	TNM stage	Follow-up (months)	Claudin-1 expression	Detection method	Antibody	Cutoff value	High expression	Outcome	NOS (score)	Data extraction method
Karabulut	2015	Turkey	140	Colorectal	60.0	I-IV	14.0	Serum	ELISA	YHB0737HU	>8.4 ng/ml	50.0%	OS/DFS	7	K-M method
Matsuoka	2011	Japan	156	Colorectal	65.0	II-IV	79.0	Protein	IHC	E3411	PP > 33%	20.5%	OS/DFS	8	Univariate
Nakagawa	2011	Japan	119	Colorectal	NR	I-IV	46.8	mRNA	RT-PCR	NR	PP > median	50.0%	OS/DFS	8	K-M method
Resnick	2005	USA	129	Colon	72.5	II	96.0	Protein	IHC	Polyclonal rabbit	SI ≥ 2	24.8%	OS/DFS	9	K-M method
Shibutani	2013	Japan	344	Colorectal	66.8	II-III	51.7	Protein	IHC	Polyclonal rabbit	PP > 25%	68.0%	OS/DFS	9	K-M method
Singh	2011	USA	250	Colon	64.6	I-IV	46.1	Protein	IHC	Anti-claudin1	>median	50.0%	OS	6	K-M method
Yoshida	2011	Japan	306	Rectum	64.0	II-III	38.0	Protein	IHC	Monoclonal antibodies	PP > 30%	46.7%	OS/DFS	8	K-M method
Kim	2019	Korea	260	Colon	63.5	I-IV	NR	Protein	IHC	Anti-claudin-1	ISS ≥ 6	57.3%	OS/DFS	8	K-M method

NR: not reported; IHC: immunohistochemistry; RT-PCR: reverse transcriptase polymerase chain reaction; SI: staining intensity; PP: positive cell percentage; immunostaining score (ISS) = PP∗SI; OS: overall survival; DFS: disease-free survival; K-M: Kaplan-Meier; NOS: Newcastle Ottawa Scale.

**Table 2:** Meta-analysis of the correlation between claudin-1 expression and clinicopathological factors of colorectal cancer.

Clinicopathological parameter	No. of studies	Participants	RR (95% CI)	Analysis model	Heterogeneity		Test for overall effect	
					*I* ^2^ (%)	*P* value	*Z* test	*P* value
Tumor type (poorly vs. well)	6	1312	0.60 (0.49, 0.73)	Fixed	33	0.19	4.94	<0.00001
Venous invasion (+ vs. -)	4	1029	0.81 (0.70, 0.95)	Fixed	0	0.40	1.59	0.0010
Lymphatic invasion (+ vs. -)	4	1028	0.83 (0.74, 0.92)	Fixed	0	0.42	3.32	0.0009
Perineural invasion (+ vs. -)	2	566	0.57 (0.25, 1.31)	Random	80	0.03	1.33	0.18
Depth of invasion (T3, 4 vs. T1, 2)	4	1029	0.94 (0.72, 1.22)	Random	70	0.02	0.47	0.64
Lymph node metastasis (+ vs. -)	4	1029	0.91 (0.82, 1.02)	Fixed	0	0.60	1.66	0.10
Distant metastasis (+ vs. -)	2	379	0.88 (0.67, 1.15)	Fixed	0	0.50	0.94	0.35
TNM stage (III, IV vs. I, II)	3	722	0.75 (0.54, 1.04)	Random	69	0.04	1.71	0.09
Size (larger vs. smaller)	2	275	0.88 (0.60, 1.29)	Fixed	0	0.68	0.65	0.52
Gender (male vs. female)	5	1185	1.02 (0.92, 1.14)	Fixed	0	0.44	0.43	0.66
Age (older vs. younger)	2	275	1.15 (0.83, 1.60)	Fixed	0	0.50	0.97	0.33
Tumor site (colon vs. rectum)	2	500	1.01 (0.87, 1.17)	Fixed	0	0.81	0.10	0.92

RR: risk ratio; Random, random effects model; Fixed: fixed effect model.

**Table 3:** Metaregression analysis for OS and DFS.

Covariates	Multivariate analysis (OS)			Multivariate analysis (DFS)		
	Coefficient	SE	*P* value	Coefficient	SE	*P* value
Site	-0.206	0.518	0.717	-0.522	0.181	0.213
TNM stage	0.039	0.435	0.934	0.121	0.133	0.531
Detection method	0.167	0.758	0.840	0.066	0.315	0.868
NOS score	-0.573	0.809	0.530	-0.638	0.335	0.308
Data extraction method	0.344	0.513	0.550	-1.313	0.550	0.253

## Supplementary Materials

Supplementary materials The supplementary material showed the quality assessment of the included studies by Newcastle Ottawa Scale (NOS). The NOS scores range from 0 to 9, and studies scoring out of 6 were considered high quality.
