# Corrigendum to “Claudin-1 Is a Valuable Prognostic Biomarker in Colorectal Cancer: A Meta-Analysis”

**DOI:** 10.1155/2020/9328192

**Published:** 2020-10-16

**Authors:** Didi Zuo, Jiantao Zhang, Tao Liu, Chao Li

**Affiliations:** ^1^Department of Endocrinology and Metabolism, The First Hospital of Jilin University, Changchun, China; ^2^Department of Colorectal and Anal Surgery, The First Hospital of Jilin University, Changchun, China

In the article titled “Claudin-1 Is a Valuable Prognostic Biomarker in Colorectal Cancer: A Meta-Analysis” [1], Dr. Guang Ning is to be removed from the author list. All authors have agreed with the updated author list, which is shown above.
