# Supplementary material for: Claudin-1 Is a Valuable Prognostic Biomarker in Colorectal Cancer: A Meta-Analysis
Source: Gastroenterol Res Pract. 2020 Aug 14;2020:4258035. doi: 10.1155/2020/4258035 (PMC7443231; doi:10.1155/2020/4258035)
Supplement: Supplementary materials — The supplementary material showed the quality assessment of the included studies by Newcastle Ottawa Scale (NOS). The NOS scores range from 0 to 9, and studies scoring out of 6 were considered high quality. [file 4258035.f1.doc]

**Table S1** Quality assessment of the included studies.

| First author | Year | Selection^1^ | | | | Comparability^2^ | Outcome^3^ | | |  |
| --- | --- | --- | --- | --- | --- | --- | --- | --- | --- | --- |
|  |  | Representativeness | Selection of | Ascertainment | No primary outcome | Comparable on | Outcome | Adequate | Loss to | Total |
|  |  | of exposed cohort | non-exposed | of exposure | was present at start | confounder | Assessment | follow-up | follow-up | Score |
|  |  | ★ | cohort | ★ | of study | ★★ | ★ | ★ | ★ |  |
|  |  |  | ★ |  | ★ |  |  |  |  |  |
| Karabulut | 2015 | 1 | 1 | 1 | 1 | 1 | 1 | 1 | 0 | 7 |
| Matsuoka | 2011 | 1 | 1 | 1 | 1 | 1 | 1 | 1 | 1 | 8 |
| Nakagawa | 2011 | 1 | 1 | 1 | 1 | 2 | 1 | 1 | 0 | 8 |
| Resnick | 2005 | 1 | 1 | 1 | 1 | 2 | 1 | 1 | 1 | 9 |
| Shibutani | 2013 | 1 | 1 | 1 | 1 | 2 | 1 | 1 | 1 | 9 |
| Singh | 2011 | 1 | 0 | 1 | 1 | 1 | 1 | 1 | 0 | 6 |
| Yoshida | 2011 | 1 | 1 | 1 | 1 | 1 | 1 | 1 | 1 | 8 |
| Kim | 2019 | 1 | 1 | 1 | 1 | 2 | 1 | 1 | 0 | 8 |

^1^ “Selection” defined as representativeness of cases, selection of controls, exposure ascertainment, and no death when investigation began.

^2^ “Comparability” defined as comparable on confounders.

^3^ “Outcome” defined as outcome assessment, adequate follow-up, and loss to follow-up rate.
